# Fatal Spotted Fever Rickettsiosis, Kenya

**DOI:** 10.3201/eid1005.030537

**Published:** 2004-05

**Authors:** Jeremiah S. Rutherford, Kevin Macaluso, Nathaniel Smith, Sherif R. Zaki, Christopher D. Paddock, Jon Davis, Norman Peterso, Abdu F. Azad, Ronald Rosenberg

**Affiliations:** * United States Army Medical Research Unit, Nairobi, Kenya; † University of Maryland School of Medicine, Baltimore, Maryland, USA; ‡ Kijabe African Inland Church Mission Hospital, Kijabe, Kenya; § Centers for Disease Control and Prevention, Atlanta, Georgia, USA

**Keywords:** *Rickettsia*, Kenya, tick typhus, African tick-bite fever

## Abstract

We report a fatal case of rickettsiosis in a woman from the United States living in Kenya, who had a history of tick exposure. Immunohistochemical staining of skin, kidney, and liver demonstrated spotted fever group rickettsiae. The clinical findings, severity, and fatal outcome are most consistent with *Rickettsia conorii* infection.

*Rickettsia* are gram-negative, obligate intracellular bacteria that are able to invade various eukaryotic host cells such as reticuloendothelial cells and vascular endothelial cells in vertebrates, and epithelial cells in their invertebrate arthropod vectors. Rickettsiae are transmitted to humans through infected arthropods, and hard ticks serve as both vectors and reservoir hosts for most spotted fever group (SFG) rickettsiae (*1*).

Two SFG rickettsiae are most commonly identified in Africa, *Rickettsia conorii* and *R. africae*. *R. conorii*, the etiologic agent of Kenyan tick typhus or more commonly known as Mediterranean spotted fever, is transmitted predominately through the bite of infected *Rhipicephalus* ticks; it has been reported in Central and South Africa (*2*). *R. africae*, the causative agent of African tick bite fever, is transmitted by *Amblyomma* ticks and has also been reported in South and Central Africa, including Kenya (*3*,*4*). *R. africae* has also been identified in 119 travelers who had recently visited South Africa, Swaziland, Lesotho, Gambia, Tanzania, Kenya, Gabon, and Côte d’ Ivoire (*5*). A third SFG rickettsia, *R*. *aeschlimannii*, has recently been described in South Africa (*6*).

We describe a fatal case of spotted fever rickettsiosis in a missionary from the United States who lived in a rural town in the central district of Kenya, approximately 70 km north of Nairobi. Although the patient received prompt medical attention at a private hospital, her illness was initially diagnosed as malaria, and this misdiagnosis possibly contributed to the fatal outcome. This case emphasizes the need to consider spotted fever rickettsioses in the diagnosis of patients with fever in Africa, and the importance of determining the distribution and prevalence of these diseases in this region.

## Case Report

During February 1999, a 39-year-old woman sought treatment at a hospital in Kijabe, Kenya, for a “boil” on her lower left leg. Over the next 3 days, headache, myalgia, chills, sweats, and a fever of 38.5°C developed. The patient was a missionary from the United States, who frequently hiked in the forest around Kijabe with her dog. Four days before discovering the lesion on her leg, she had taken her ill dog to the local veterinarian. The dog, which was frequently infested with ticks, was diagnosed with “tick-borne fever” (an unspecified illness in dogs, associated with tick infestation) and was treated with imizol (imidocarb dipropionate). The dog’s symptoms resolved within 3 days.

Five days after the onset of her symptoms, the patient returned to the hospital when her condition had not improved. Hematologic evaluation showed a marginal decrease in leucocytes to 4,200/mm^3^ (80% polymorphonuclear cells, 2% bands, 17% lymphocytes, and 1% eosinophils), and thrombocytopenia (107,000/mm^3^). No malaria parasites were detected on thick or thin blood smears. The patient was treated with acetaminophen and released from the hospital.

The next day, the patient returned to the hospital after the onset of vomiting and a nonpuritic, blotchy, macular rash on her arms and abdomen. Because she had traveled recently to a malaria-endemic area, malaria was diagnosed. Treatment was initiated with three tablets of Fansidar (500 mg sulfadoxine and 25 mg pyrimethamine per tablet). The patient reported some improvement of symptoms that evening, and by the following day, she ceased vomiting and her fever was reduced.

On the day 9 of her illness, a generalized macular rash covered her full body, sparing her palms and soles. Her pharynx was red. Her abdomen was tender in both upper quadrants. A viral exanthem as well as malaria responding to treatment were diagnosed. Vomiting and fever began again that afternoon. That evening she became mildly confused and was admitted to Kijabe African Inland Church Mission Hospital. Her temperature was 38°C; she was tachypnic and had a nonproductive cough. She had mild epigastric tenderness, but spleen and liver margins were not palpable. An eschar surrounded by a 1.5 cm purpuric border was identified on her lower left leg. Fansidar-resistant malaria as well as a viral exanthem were diagnosed, and she was treated with intravenous fluids, quinine, acetaminophen, and promethazine. The following day, her temperature was 39.9°C, and she was unable to follow verbal commands. Multiple seizures developed, and she went into cardiopulmonary arrest. Despite attempts at cardiopulmonary resuscitation, the patient died.

**Figure 1:**
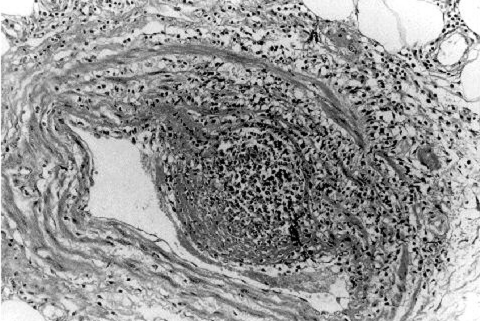
Vasculitis and edema involving medium-sized artery in the subcutaneous fat at the site of the eschar (hematoxylin and eosin stain; original magnification x100).

Histopathologic findings included extensive epidermal and dermal necrosis and acute inflammation at the site of the eschar with focal, transmural, mixed inflammatory cell infiltrates involving dermal blood vessels (Figure 1). The liver showed portal triaditis and focal eyrthophagocytosis by Kupffer cells. The spleen showed multiple microscopic necrotic foci involving red and white pulp. Multifocal lymphohistiocytic infiltrates were identified in the interstitium of the heart and kidneys. The lungs showed mild pulmonary edema and congestion.

**Figure 2:**
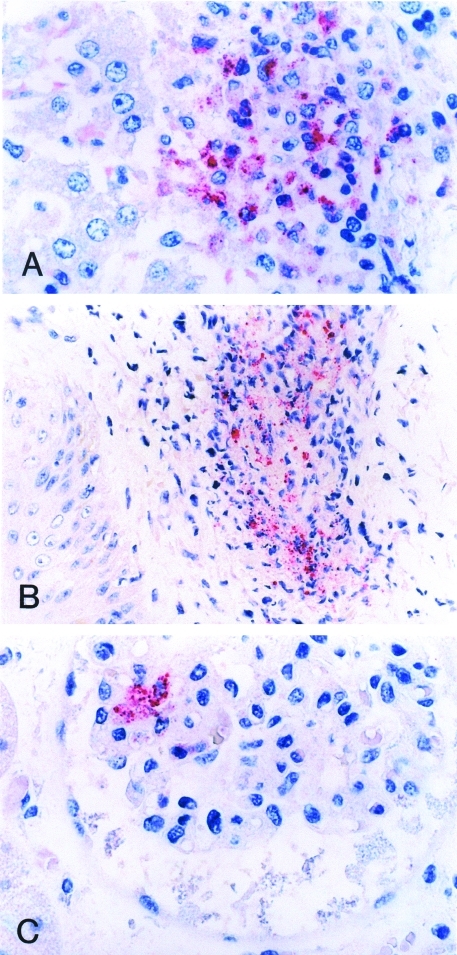
Immunohistochemical localization of spotted fever group rickettsial antigens in various tissues of a patient with fatal spotted fever rickettsiosis, by immunoalkaline phosphatase stain with naphthol phosphate–fast red substrate and hemotoxylin counterstain. Rickettsiae and rickettsial antigens (red) in Kupffer cells in liver (A), perivascular infiltrates in skin (B), and glomerular endothelium in kidney (C) (naphthol–fast red stain with hematoxylin counterstain; original magnifications x158).

Formalin-fixed, paraffin-embedded tissue and serum specimens were sent to the Centers for Disease Control and Prevention for evaluation. Immunohistochemical (IHC) staining for SFG *Rickettsia* demonstrated rickettsiae and rickettsial antigens in sections of heart, spleen, and kidney localized within and around vascular endothelial cells and reticuloendothelial cells, predominantly in areas associated with perivascular mononuclear inflammatory infiltrates (Figure 2).

A single serum sample, collected on the ninth day of illness, demonstrated neither immunoglobulin (Ig) G nor IgM antibodies reactive with *R. conorii* when tested by an indirect immunofluorescence antibody assay at a screening dilution of 1:16. To further define the causative agent, we attempted to amplify a portion of the rickettsial *rOmpA* gene, as we have done for ticks from this region (*4*), using DNA extracted from the paraffin-embedded sections of heart, spleen, and kidneys. Briefly, paraffin-embedded tissue samples were deparaffinized in xylene, and rinsed in absolute ethanol. Genomic DNA was extracted by using the Wizard genomic DNA purification kit (Promega, Madison, WI), according to the manufacturer’s protocol. Amplification of a 635-bp fragment of the *rompA* gene encoding the SFG-specific 190-kDa protein was attempted by using primers Rr190.70p (*7*) and Rr190.701 (*8*). Genomic DNA isolated from *R*. *montanensis*–infected Vero cells, and water served as positive and negative controls, respectively, for the polymerase chain reaction (PCR).

## Conclusions

Immunostaining demonstrated an SFG rickettsial infection as the cause of the patient’s death; yet, because the IHC assay reacts with several species of SFG rickettsiae (*9*), a specific etiologic agent was not determined. Serologic evaluation of a serum specimen obtained late in the course of this patient’s illness failed to demonstrate antibody reactive with SFG rickettsiae; however, some patients with fatal rickettsial infections die before notable levels of these antibodies are detected (*9*).

Additionally, repeated attempts to amplify rickettsial DNA from numerous thin-section preparations from paraffin-embedded tissues were unsuccessful. Therefore, we were unable to characterize the infecting rickettsiae using molecular techniques. Sample fixation procedures may decrease the sensitivity of the PCR, as seen in diagnostic assays for other microorganisms (*10*). Further work is needed to determine the utility of PCR as a reliable method of detecting rickettsial DNA in paraffin-embedded tissues.

The spectrum of rickettsial infections in Africa ranges from mild to severe. *R. conorii* causes a moderate to severe illness with a case-fatality rate of approximately 3%. Disease is most often characterized by a single eschar and a generalized maculopapular rash that may involve the palms and the soles (*11*). By contrast, *R. africae* causes a generally milder, self-limiting disease with fever, multiple eschars, regional lymphadenopathy, and rash in approximately 16% to 46% of patients (*3*,*12*).

Anecdotal reports and some animal and human studies suggest that sulfa-containing antimicrobial agents exacerbate the clinical severity of rickettsial infections (*13*,*14*), and the patient described here received a sulfa-containing antibiotic after malaria was diagnosed. However, an early correct diagnosis and prompt administration of effective antirickettsial therapy (e.g., doxycycline or another tertacycline) remain the primary determinants of successful clinical outcomes for patients with spotted fever rickettsioses. Evidence is accumulating that tick-borne rickettsiosis are an underreported and underappreciated cause of illness in sub-Saharan Africa. For example, while investigating this case we discovered that a mild febrile illness, accompanied by rash, developed in nearly one third of a group of boarding school students from the United States, who had camped at Masai Mara, a popular tourist destination; all had histories of tick bite (J. Rutherford, unpub. data). Additionally, recent identification of SFG rickettsiae in ticks from this area confirmed the presence of *R. africae* in *Amblyomma variegatum* collected from domesticated livestock (*4*). Rickettsial infections in Central and Sub-Saharan Africa have been reported among tourists and travelers who visited game reserves (*12*). The prevalence of spotted fever in local populations may be obscured by the lack of obvious rash and the overwhelming number of cases of malaria.

This case report highlights both an immediate and a long-term need for rickettsial surveillance and dissemination of information about rickettsioses to health care workers in Africa. In a similar manner, physicians in industrialized countries who care for ill travelers who have visited Africa should consider rickettsial infections among the differential diagnoses of febrile disease, particularly in clinical situations in which malaria has been reasonably excluded as a cause of the patient’s illness. In these contexts, further studies of the epidemiology and ecology of African SFG rickettsiae and rickettsioses are warranted.
